# Binary Solvent Extraction System and Extraction Time Effects on Phenolic Antioxidants from Kenaf Seeds (*Hibiscus cannabinus* L.) Extracted by a Pulsed Ultrasonic-Assisted Extraction

**DOI:** 10.1155/2014/789346

**Published:** 2014-01-23

**Authors:** Yu Hua Wong, Hwee Wen Lau, Chin Ping Tan, Kamariah Long, Kar Lin Nyam

**Affiliations:** ^1^Department of Food Science and Nutrition, Faculty of Applied Sciences, UCSI University, No. 1, Jalan Menara Gading, UCSI Heights, 56000 Cheras, Kuala Lumpur, Malaysia; ^2^Department of Food Technology, Faculty of Food Science and Technology, Universiti Putra Malaysia, 43400 Serdang, Selangor, Malaysia; ^3^Malaysian Agricultural Research & Development Institute (MARDI), 43400 Serdang, Selangor, Malaysia

## Abstract

The aim of this study was to determine the best parameter for extracting phenolic-enriched kenaf (*Hibiscus cannabinus* L.) seeds by a pulsed ultrasonic-assisted extraction. The antioxidant activities of ultrasonic-assisted kenaf seed extracts (KSE) were determined by a 2,2-diphenyl-1-picrylhydrazyl (DPPH) radical scavenging capacity assay, 2,2′-azino-bis(3-ethylbenzothiazoline-6-sulphonic acid) (ABTS) radical scavenging assay, **β**-carotene bleaching inhibition assay, and ferric reducing antioxidant power (FRAP) assay. Total phenolic content (TPC) and total flavonoid content (TFC) evaluations were carried out to determine the phenolic and flavonoid contents in KSE. The KSE from the best extraction parameter was then subjected to high performance liquid chromatography (HPLC) to quantify the phenolic compounds. The optimised extraction condition employed 80% ethanol for 15 min, with the highest values determined for the DPPH, ABTS, and FRAP assay. KSE contained mainly tannic acid (2302.20 mg/100 g extract) and sinapic acid (1198.22 mg/100 g extract), which can be used as alternative antioxidants in the food industry.

## 1. Introduction

Bioactive constituents with antioxidant activities have been found at high concentrations in plants [1]. Because of the potential health risks and toxicity, some synthetic antioxidants must be replaced with natural antioxidants [2]. Therefore, there is great interest in the replacement of synthetic antioxidants with natural sources, especially from plant materials [3]. Kenaf (*Hibiscus cannabinus* L.) is well known in Asia and Africa for its multipurpose use, and it has been widely cultivated in some Mediterranean areas [4]. According to Cheng et al. [5], the traditional use of this plant is focused on fibre production, such as making ropes, sacks, canvases, and carpets. Keshk et al. [6] showed that new kenaf applications have been developed for the pulp and paper industry, oil adsorption and potting media, board making, filtration media, and animal feed. The kenaf seeds are considered a waste product, and they are used mainly for animal feeds in the cattle, sheep, camel, and poultry industries. Using this waste is very important for kenaf cultivation and for increasing grower income. In addition, this plant was also composed of various active components, which have long been prescribed in traditional folk medicine from Africa and India [7]. According to Nyam et al. [8], there were seven main phenolic compounds identified in the kenaf seed oil, namely, gallic acid, p-hydroxybenzoic acid, caffeic acid, vanillic acid, syringic acid, p-coumaric, and ferulic acid. Increasing interest in replacing synthetic antioxidants has led to investigations of natural antioxidant sources, especially in plant materials.

Ultrasound-assisted extraction provides a mechanical effect, allowing greater solvent penetration into the sample matrix, increasing the contact surface area between the solid and liquid phase and, as a result, the solute quickly diffuses from the solid phase to the solvent [9, 10]. Kenaf seed oil has normally been extracted by Soxhlet extraction [11] and supercritical carbon dioxide fluid extraction [12]. However, there have been limited studies on the extraction of kenaf seed extract. Yusri et al. [13] have extracted phenolic compounds from kenaf seed by normal solvent extraction. There is no information regarding the ultrasonic-assisted extraction of kenaf seeds. Therefore, pulse ultrasound-assisted extraction (PUAE) was chosen to extract kenaf seeds in this study. PUAE required lower electrical energy consumption, high extraction time reduction, higher antioxidant yield, and increased antioxidant activity.

The objectives of this study were to determine the best parameters (ethanol concentration and extraction time) for extracting phenolic-enriched kenaf seeds by pulsed ultrasonic-assisted extraction (PUAE), to compare the antioxidant properties among different extraction parameters, and to determine the phenolic compounds in kenaf seed extract, which were extracted by using the best parameters.

## 2. Materials and Methods

### 2.1. Sample

Dried kenaf (*Hibiscus cannabinus* L.) seed was obtained from the Malaysian Agricultural Research and Development Institute (MARDI, Serdang, Malaysia) and was ground into powder with a grinder (SHARP, Japan). The kenaf seed powder particle size was 1 mm.

### 2.2. Sample Extraction

#### 2.2.1. Pulsed Ultrasound-Assisted Extraction (PUAE)

Nine 500 mL Scott bottles were prepared. Each set of three bottles was filled with 60%, 80%, and 100% ethanol. Fifty grams of ground kenaf seed was added to the solvent to undergo ultrasonic extraction (Ultrasonic Homogeniser Labsonic P, 400 W, Sartorius, AG) with a 5 min pulse duration period and 5 min pulse interval period. A 5 min pulse duration period and 5 min pulse interval period were considered as 1 extraction cycle. Extractions were carried out with 1 extraction cycle, 2 extraction cycles, and 3 extraction cycles for each ethanol concentration. The resulting kenaf seed extract was centrifuged at 3500 rpm for 10 min. The supernatant of the kenaf seed extract was collected and underwent filtration and the pellet was discarded. The filtered extract was evaporated by a rotary evaporator (Rotavapor R-200, Buchi, Switzerland) and left in the centrifuge tube, which was wrapped with aluminium foil.

### 2.3. Antioxidant Activities

#### 2.3.1. 2,2-Diphenyl-1-picrylhydrazyl (DPPH) Radical Scavenging Capacity Assay

The 2,2-diphenyl-1-picrylhydrazyl (DPPH) radical scavenging capacity assay for all nine kenaf seed extracts was determined according to Liu et al. [14] with slight modifications. The kenaf seed extract (200 *μ*L) with concentration of 1 mg/mL was added to 2.8 mL of ethanol and 1 mL of 0.004% of DPPH solution. After 30 min of incubation, the absorbance was measured against a blank reagent (ethanol) at 517 nm by using a UV-Vis spectrophotometer (Model XTD 5, Secomam, Domont, France). All DPPH radical scavenging activities of the KSE were expressed as percentage inhibition. Inhibition percentage was calculated by using the formula below:

(1) $$\begin{array}{c} \text{Inhibition}\;\text{percentage}\;\left(\text{IP}\right)=\frac{\left(\text{blank}-\text{KSE}\right)}{\text{blank}}\times 100, \end{array}$$

where blank and KSE were the absorbance values of the blank and the KSE.

#### 2.3.2. 2,2′-Azino-bis(3-ethylbenzothiazoline-6-sulphonic Acid) (ABTS) Radical Scavenging Assay

ABTS was carried out according to Floegel et al. [15] with slight modifications. ABTS (7 mM) and 2.45 mM of potassium persulphate (K_2_O_8_S_2_) were mixed and kept in the dark at room temperature for 12–16 h for activation. The activated mixture was then adjusted with ethanol at 734 nm to 0.7 AU (±0.02). Fifty milliliters of the sample was added to 1950 *μ*L of ABTS and allowed to react for 6 min. The absorbance at 734 nm was measured by using a UV-Vis spectrophotometer (Model XTD 5, Secomam, Domont, France) against a blank reagent.

All ABTS radical scavenging activities of the KSE were expressed as percentage inhibition. Inhibition percentage was calculated by using the formula below:

(2) $$\begin{array}{c} \text{Inhibition}\;\text{Percentage}\left(\text{IP}\right)=\frac{\left(\text{blank}-\text{KSE}\right)}{\text{blank}}\times 100, \end{array}$$

where blank and KSE are the absorbance values of the blank and the KSE.

#### 2.3.3. *β*-Carotene Bleaching (BCB)

The *β*-carotene bleaching activity of the extracts was evaluated by the *β*-carotene linoleate model system [16]. A solution of *β*-carotene was prepared by dissolving *β*-carotene (5 mg) in chloroform (50 mL). Three milliliters of this solution was pipetted into a round-bottom flask. Then, the chloroform was removed at 40°C under vacuum. The linoleic acid (40 mg), Tween 20 emulsifier (400 mg), and distilled water (100 mL) were added to the flask with vigorous shaking. An aliquot (3 mL) of this emulsion was transferred into different test tubes containing different concentrations of the extracts (0.1 mL). The tubes were shaken and incubated at 50°C for 30 min in a water bath. As soon as the emulsion was added to each tube, the zero time absorbance was measured at 470 nm using a UV-Vis spectrophotometer (Model XTD 5, Secomam, Domont, France). The BCB antioxidant activity was expressed as inhibition percentage with reference to the control after 30 min standardized incubation time with the following formula:

(3) $$\begin{array}{c} \%\text{AA}=100\times \frac{\left({\text{DR}}_{\text{control}}-\;{\text{DR}}_{\text{sample}}\right)}{{\text{DR}}_{\text{control}}}, \end{array}$$

where AA is the antioxidant activity; DR_control_ is the degradation rate of the control = [(*a*/*b*)/30]; DR_sample_ is the degradation rate in the presence of the sample = [(*a*/*b*)/30], where *a* is the absorbance at zero time (first result recorded) while *b* is the absorbance at 30 min (second result recorded).

#### 2.3.4. Ferric Reducing Antioxidant Power (FRAP)

FRAP was carried out according to Wootton-Beard et al. [17] with slight modifications. The FRAP reagent was prepared by mixing 25 mL of acetate buffer with 2.5 mL of 2,4,6-tripyridyl-s-triazine (TPTZ) and 2.5 mL of FeCl_3_·6H_2_O. The mixture was maintained in a 37°C water bath (Lab Companion, Korea). Fifty milliliters of the sample was added to 950 *μ*L of FRAP reagent in a test tube. The mixture was allowed to react for 30 min in the dark at room temperature. The absorbance was measured at 593 nm by using UV-Vis spectrophotometer (Model XTD 5, Secomam, Domont, France) against a blank reagent after 30 min. The calibration equation for Trolox was *y* = 1.5535*x* + 0.4198. The results were expressed in milligrams of Trolox/100 g of kenaf seed extract.

#### 2.3.5. Total Phenolic Content (TPC)

The total phenolic content of the kenaf seed extract was determined using Folin-Ciocalteu assay, based on the method described by Lim et al. [18] with slight modifications. A kenaf seed extract (300 *μ*L) of 10 mg/mL was added to 1.5 mL of Folin-Ciocalteu reagent (FCR) and mixed well. The mixture was allowed to stand at room temperature for 5 min. Then, 1.2 mL of sodium carbonate (7.5%, w/v) solution was added and mixed thoroughly by using a vortex mixer and allowed to stand for 30 min. After 30 min, the absorbance was measured against a blank reagent (ultrapure water mixed with FCR and sodium carbonate) at 765 nm using a UV-Vis spectrophotometer (Model XTD 5, Secomam, France). The calibration equation for gallic acid was *y* = 8.525*x* + 0.0331 (*r* ^2^ = 0.9989). The total phenolic content was expressed in milligrams of gallic acid/100 g of kenaf seed extract. The results were expressed in milligrams of gallic/100 g of kenaf seed extract.

#### 2.3.6. Total Flavonoid Content (TFC)

The total flavonoid concentration of the kenaf seed extract was measured by using a colorimetric assay in accordance with the method of Verzelloni et al. [19] with slight modifications. A kenaf seed extract (250 *μ*L) of 10 mg/mL was transferred into a test tube. One millilitre of deionised water and 150 *μ*L of 150 mg/mL sodium nitrite were added to the test tube and allowed to stand for 6 min. Then, 75 *μ*L aluminium chloride (AlCl_3_) (100 mg/mL) was added. The test tubes were allowed to stand for 5 min, then 1 mL of 40 mg/mL sodium hydroxide (NaOH) was added. Finally, 25 *μ*L of deionised water was added for a final volume of 2.5 mL. The absorbance was measured at 510 nm using a UV-Vis spectrophotometer (Model XTD 5, Secomam, France) against a blank reagent. The calibration equation for catechin was *y* = 2.895*x* − 0.0185 (*r* ^2^ = 0.9956). The results were expressed in milligrams of catechin/100 g of kenaf seed extract.

### 2.4. Phenolic Compound Quantification

The phenolic compounds in the kenaf seed extract were quantified by HPLC, as described by Baydar et al. [20] with a slight modification. The HPLC system was equipped with a diode array detector (DAD). Phenolic compound separation was carried out using a reversed-phase HPLC column (Purospher star 5 *μ*m × 250 mm × 4.6 mm, Merck, Germany). The column temperature and detection wavelength were set at 30°C and 210 nm, respectively. A gradient elution system of solvent A (water with 0.1% phosphoric acid) and solvent B (methanol with 0.1% phosphoric acid) was used (5% B (0 min); 50% B (5 min); 55% B (65 min); and 5% B (70 min)). The flow rate was 1 mL/min, and the injected volume was 20 *μ*L. The phenolic compound chromatographic peaks were confirmed by comparing their retention times with those of the reference standards. Gallic acid, tannic acid, catechin hydrate, 4-hydroxybenzaldehyde, 4-hydroxybenzoic acid, syringic acid, sinapic acid, ferulic acid, naringin, and protocatechuic acid were used as phenolic compound standards.

### 2.5. Statistical Analysis

All experiments and/or measurements were duplicated. Analysis of variance (ANOVA) was carried out and the average values were compared with Fisher's multiple comparison test. All statistical analyses were performed using Minitab 13 for Windows (Pennsylvania, United States).

## 3. Results and Discussion

### 3.1. Extraction Solvent Evaluation

In the present study, an extraction using a binary ethanol and water solvent was adopted after considering health and safety in handling [21]. This experimental result was in accordance with previous reports, suggesting that a binary solvent system was superior to a monosolvent system (water or pure ethanol) in the extraction of phenolic compounds with regard to their relative polarity [22, 23]. The solubility of phenolic compounds depends on the chemical nature of the plant tissue and the polarity of the solvent system [24]. Ethanol (60%) was chosen as the lowest solvent concentration in this study. This solvent was chosen because previous studies showed that antioxidant compounds from plant sources were best extracted by 70%–80% ethanol concentrations [25–27]. In this study, the kenaf seeds extracted by 80% ethanol had better 2,2-diphenyl-1-picrylhydrazyl (DPPH) radical scavenging capacity assay results than the 100% ethanol kenaf seed extract (Table 1). This circumstance may be explained by the inability of 100% ethanol to extract phenolic compounds, which are more water-soluble (hydrophilic). The presence of water in the extraction eases the release of hydrophilic antioxidants. Trabelsi et al. [28] showed that the addition of 20% water to methanol, acetone, or ethanol can enhance the extraction of antioxidants from *Limoniastrum monopetalum* leaves.

DPPH radical scavenging capacity assay is frequently used to determine the free radical scavenging ability of various food components [29, 30]. In addition, the 2,2′-azino-bis(3-ethylbenzothiazoline-6-sulphonic acid) (ABTS) radical scavenging assay is also used to study the free radical scavenging ability of a sample by using a moderately stable nitrogen-centred radical species [31]. DPPH and ABTS both yielded consistent results in the measurement of antioxidant activity [32]. Therefore, DPPH and ABTS were chosen to determine the best extraction parameters. In this study, the 80% ethanol and 15 min condition were the optimised method for kenaf seed extraction. In addition, ferric reducing antioxidant power (FRAP) and *β*-carotene bleaching assays (BCB) were also in accordance with this observation, in which the highest value in each analysis was identified. However, the antioxidant activities indicated by the *β*-carotene bleaching assay were relatively lower than those of the DPPH, ABTS, and FRAP assays. The *β*-carotene bleaching test was carried out in a state of emulsion. The result was based on the fading of the characteristic yellow *β*-carotene colour, which was caused by its reaction with radicals formed from the oxidation of linoleic acid. Therefore, it can be deduced that the hydrophilic antioxidant content is higher compared to hydrophobic antioxidants in kenaf seed extract.

In this study, the optimised condition (80% ethanol for 15 min) that produced the highest antioxidant content for kenaf seed extract did not give the optimised results for the total phenolic content (TPC) and total flavonoid content (TFC) assays. Based on the present experimental results, it was predicted that kenaf seed contained diverse phenolic compounds with different polarities. Thus, no single ethanol concentration was able to recover all of the individual phenolic compounds from the samples. Durling et al. [33] suggested that the TPC, which had its highest value at low ethanol concentrations, contained a higher proportion of hydrophilic compounds. The TFCs recovered at high ethanol concentrations were lipophilic compounds. Additionally, a high individual phenolic compound yield will not necessarily be associated with a high antioxidant capacity, which, indeed, is dependent on the synergistic effects of the extracted phenolics. Therefore, DPPH and ABTS analyses were more suitable for choosing the optimised parameter.

### 3.2. Extraction Time Evaluation

The DPPH and ABTS assays showed increasing scavenging activity trends when the extraction time increased at all ethanol concentrations (Table 1). However, the total phenolic content (TPC) and total flavonoid content (TFC) for kenaf seeds extracted with 100% ethanol decreased as the extraction time increased. This trend can be explained by the potential extraction time increase by the loss of antioxidants following heat or oxygen exposure [34]. The decrease of antioxidant activity and phenolic content only appeared in 100% ethanol concentration extraction but did not appear in 60% and 80% ethanol concentration extraction when the extraction time increased from 5 to 15 min. The maximum temperature reached for 60% and 80% ethanol concentration extraction after 15 min was lower than 70°C while the temperature of 100% ethanol increases rapidly during the extraction and reached a temperature above 70°C. It is generally believed that most of the antioxidant compounds have shown rapid degradation when the temperature was above 70°C [24]. In a complex polyphenol system, a temperature increase can promote molecular collisions, favouring polymerisation and reducing antioxidant capacity [35]. The difference in optimum extraction times for TPC and TFC may be explained by different degrees of phenolic polymerisation, the phenolic compound solubility, and their interactions with other food constituents, which then led to different time requirements for reaching the equilibrium between the solution in the solid matrix (*M. citrifolia*) and in the bulk solution (ethanol) [36].

This study showed that the optimum extraction time for antioxidant compounds varies with the antioxidant capacity. This phenomenon postulated that the estimation of ABTS and DPPH radical-scavenging capacities is not solely dependent on a single group of antioxidant compounds. ABTS and DPPH radical-scavenging capacities are based on the capacity of existing compounds to scavenge ABTS or DPPH radicals [37]. These data highlighted the impact of the solvent choice on the extraction efficiency. Other studies have shown that the ethanol concentration, extraction time, and temperature affect the recovery of phenolic compounds and the antioxidant capacity of plant extracts [38].

### 3.3. Phenolic Compounds

The HPLC analysis of phenolic compounds has become one of the most dominant analytical procedures because of its advantages. The advantages of HPLC are simple sample treatment, possible preseparation and impurity removal, capacity to change the polarity of the mobile phase during analysis, and high reproducibility. In this study, the wavelength of the detector was set as 210 nm since the maximum absorbance for most of phenolic compounds is at 210 nm. Moreover, the research done by Du and Chen [39] showed that, at wavelength below 210 nm, the baseline becomes unstable because of the strong absorption of methanol, which caused the interferences to the detection, while at wavelength above 215 nm, the adsorption of analytes diminished significantly. The phenolic compounds in kenaf seed extract were extracted with 80% ethanol for 15 min and determined by HPLC-DAD (Table 2), revealing the presence of gallic acid, tannic acid, catechin, benzaldehyde, benzoic acid, syringic acid, sinapic acid, ferulic acid, naringin acid, and protocatechuic acid, as some of the phenolic compounds that were found in kenaf seed oil [40].

Tannic acid (2302.20 mg/100 g extract) and sinapic acid (1198.22 mg/100 g extract) were the major compounds in kenaf seed extract. Tannic acid has been shown to possess antioxidant, antimutagenic, and anticarcinogenic properties [41]. Sinapic acid is a cinnamic acid derivative, and it possesses 3,5-dimethoxy and 4-hydroxyl substitutions in the phenyl group of cinnamic acid. Sinapic acid is widely distributed in the plant kingdom and can be obtained from various sources, such as rye, broccoli, cabbage, and kale [42]. Sinapic acid has already been pharmacologically evaluated for its potent antioxidant, anxiolytic, anti-inflammatory, and peroxynitrite scavenging effects and neuroprotective effects [43]. Apart from that, Nyam et al. [44] proved that kenaf seed extract showed greater effect than synthetic antioxidant (BHA) in prevention of sunflower oil oxidation. Therefore, we suggested that kenaf seed extract can be used as an alternative to synthetic antioxidants for the efficient, large-scale production of tannic acid and other high-value secondary metabolites.

## 4. Conclusion

This study showed that kenaf seeds extracted with 80% ethanol for 15 min had the highest DPPH, ABTS, and FRAP assay values. Therefore, the best compromise between high antioxidant compound extraction yield and cost as well as practicality was found for a 15 min extraction in 80% for the kenaf seed extract. It was also found that the kenaf seed extract primarily contained tannic acid and sinapic acid, which can be used as alternative antioxidants in the industrial sector.

## Figures and Tables

**Table 1:** 2,2-Diphenyl-1-picrylhydrazyl (DPPH) radical scavenging capacity, 2,2^'^-azino-bis(3 ethylbenzothiazoline-6-sulphonic acid) (ABTS) radical scavenging assay, total phenolic content (TPC), total flavonoid content (TFC), ferric reducing antioxidant power (FRAP), and *β*-carotene bleaching (BCB) assays of kenaf seed extract extracted by different ethanol concentrations and extraction times.

Extraction parameter		DPPH (%)	ABTS (%)	TPC (mg GAE/100 g)	TFC (mg CE/100 g)	FRAP (mg TEAC/100 g)	BCB (%)
Ethanol concentration	Time (min)						
60%	5	28.71 ± 0.55^i^	12.86 ± 0.34^e^	1579.94 ± 56.23^cd^	592.40 ± 4.89^h^	1011.91 ± 34.46^i^	28.93 ± 0.68^c^
	10	35.04 ± 0.62^g^	23.54 ± 0.33^d^	1757.65 ± 27.80^b^	666.67 ± 4.46^g^	1137.43 ± 37.35^h^	31.07 ± 2.94^abc^
	15	42.82 ± 0.33^e^	47.02 ± 0.99^c^	1913.08 ± 2.14^a^	849.74 ± 4.46^f^	1299.97 ± 21.27^g^	33.56 ± 0.70^a^
80%	5	52.89 ± 0.70^c^	45.33 ± 1.49^c^	1407.21 ± 13.25^f^	1670.98 ± 10.69^e^	3554.55 ± 86.52^c^	28.87 ± 0.58^c^
	10	60.35 ± 0.35^b^	55.44 ± 1.45^b^	1506.92 ± 7.38^e^	2394.65 ± 4.35^d^	4421.95 ± 39.46^b^	34.22 ± 0.54^a^
	15	66.68 ± 0.38^a^	69.78 ± 0.66^a^	1538.59 ± 13.25^d^	2705.53 ± 4.90^c^	5661.09 ± 29.91^a^	33.46 ± 1.10^ab^
100%	5	32.07 ± 0.87^h^	8.72 ± 0.32^g^	1233.31 ± 9.51^h^	4082.04 ± 3.31^a^	3247.18 ± 21.27^d^	31.96 ± 0.40^b^
	10	39.68 ± 0.76^f^	11.46 ± 0.39^f^	1594.31 ± 8.32^c^	2836.79 ± 5.90^b^	1570.33 ± 16.09^e^	18.35 ± 0.76^d^
	15	46.94 ± 0.57^d^	12.62 ± 0.20^e^	1278.77 ± 16.63^g^	2393.78 ± 4.46^d^	1402.96 ± 28.49^f^	15.86 ± 0.51^e^

Value are presented in means ± standard deviation (*n* = 4); mean values at the same column with different superscripts are significantly different (*P* < 0.05).

GAE: gallic acid equivalents.

CE: catechin equivalents.

TEAC: Trolox equivalent.

**Table 2:** Phenolic compounds in kenaf seed extract extracted by 80% ethanol for 15 min.

Phenolic compounds	Concentration (mg/100 g)
Gallic acid	123.36 ± 0.31
Tannic acid	2302.20 ± 16.48
Catechin hydrate	502.73 ± 1.98
4-Hydroxybenzaldehyde	116.14 ± 7.87
4-Hydroxybenzoic acid	255.84 ± 7.74
Syringic acid	61.56 ± 5.97
Sinapic acid	1198.22 ± 21.82
Ferulic acid	288.38 ± 14.05
Naringin	43.79 ± 3.19
Protocatechuic acid	170.83 ± 8.22

Total	5880.56

Value are presented in means ± standard deviation (*n* = 4).
